# A systematic review of general surgery robotic training curriculums

**DOI:** 10.1016/j.heliyon.2023.e19260

**Published:** 2023-08-18

**Authors:** Haydee Del Calvo, Min P. Kim, Ray Chihara, Edward Y. Chan

**Affiliations:** a Division of Thoracic Surgery, Department of Surgery, Houston Methodist Hospital, Houston, TX, USA; b Department of Surgery and Cardiothoracic Surgery, Weill Cornell Medical College, Houston Methodist Hospital, Houston, TX, USA

**Keywords:** Robotic surgery, Resident curriculum, Curriculum assessment, Systematic review

## Abstract

**Background:**

As of the most recent surveys of resident programs in 2018, only slightly more than half of programs have formal robotic training curriculums implemented. Fewer programs have further assessed their own curriculum and its benefit.

**Method:**

We conducted a PubMed/MEDLINE literature search for robotic surgery curriculums and those that had assessment of their programs.

**Results:**

A total of 11 studies were reviewed. When reviewed in chronological order, there has been a progression towards more robotic specific objective data analysis as opposed to subjective surveying. There is a wide variation in curriculums, but simulation use is pervasive.

**Conclusions:**

Our review makes evident two important concepts-there is great variety in training curriculums and there is great benefit in implementation. The importance is in establishment of what makes resident training effective and supports the adaptable and successful surgeon. This may come from an adaptable curriculum but a structured test-out assessment.

## 1 Introduction

Robotic surgical systems, most commonly the da Vinci platform (Intuitive Surgical, Sunnyvale, CA), have now reached a new milestone of surgeon use in more than 10 million procedures [1]. The systems are accounted to be about 6500 around the world. This integration into the surgical sphere has not only attracted the attention of surgeons worldwide, but now it is also a sought-after component of surgery that patients ask for and expect. This rapid growth has taken place mostly in the last two decades. In the year 2000 the Food and Drug Administration approved the da Vinci system for use in general surgery and since then, it has been adopted by numerous surgical specialties [2]. The first group to significantly integrate this system into their practice was the urologists, where radical prostatectomies soon began gaining rapid acceptance. They were closely followed by gynecologists for hysterectomies. The technological advancements that the new systems introduced, such as 3D high-definition image and 360-degree range of motion, were initially used mainly in these surgical specialties that operated in difficult anatomical regions such as the pelvis. Now the benefits of the robotic system have been more acknowledged and appreciated leading to incorporation into additional surgical specialties. A cohort study of 73 hospitals in Michigan from 2012 to 2018 found that the use of robotic surgery for general surgery procedures increased 8.4-fold from 1.8% to 15.1%. It is also important to note that within the same time span, both laparoscopic and open surgery use declined [3]. As robotic-assisted surgery has become a pervasive tool in the surgical arena, resident training has continued to be greatly impacted by this technological introduction.

An anonymous, national, web-based survey of residents in Accreditation Council for Graduate Medical Education (ACGME) accredited general surgery resident training programs in 2013 showed that of the responding residents, 96% noted the availability of robotic systems at their institution but only 63% indicated participation in robotic surgical cases [4]. In this same study, 60% of responding residents noted no training prior to first robotic case and 64% of residents indicated that they believed formal training would be of value. Five years later, in a similar study surveying ACGME programs, it was found that in 2018 only 68% of responding programs had formal robotic training for residents [5]. So now that we know the value of these systems and the pervasive nature of robotic-assisted surgery, why are we not integrating it further into training? This review aims to gather the information that we currently have on surgical training robotic curriculums and the assessment measures that are being used. While there have been reviews of the literature to assess the curricula currently available to resident programs by different organizations, with this study we took it one step further to gather information on programs that have decided to incorporate a robotics curriculum and have done studies to assess their progress. It provides an objective view of how programs have implemented the need to train residents within a new technology platform and therefore allows readers to assess implementation feasibility. Robotic technology, like laparoscopic surgery, is a new skill and it should be taught in the same structured ways. Currently, we have programs like Fundamentals of Laparoscopic Surgery to provide education and assessment. As programs continue to develop their curriculums, we find trends in the key elements for resident education of the robotic technology. This is what we aim to show with our systematic review. With this information of implementation trends and key components we hope to show the richness in variety and value in incorporation that has currently been performed and therefore the value and feasibility in standardizing the implementation of a curriculum.

## 2 Methods

**Fig. 1:**
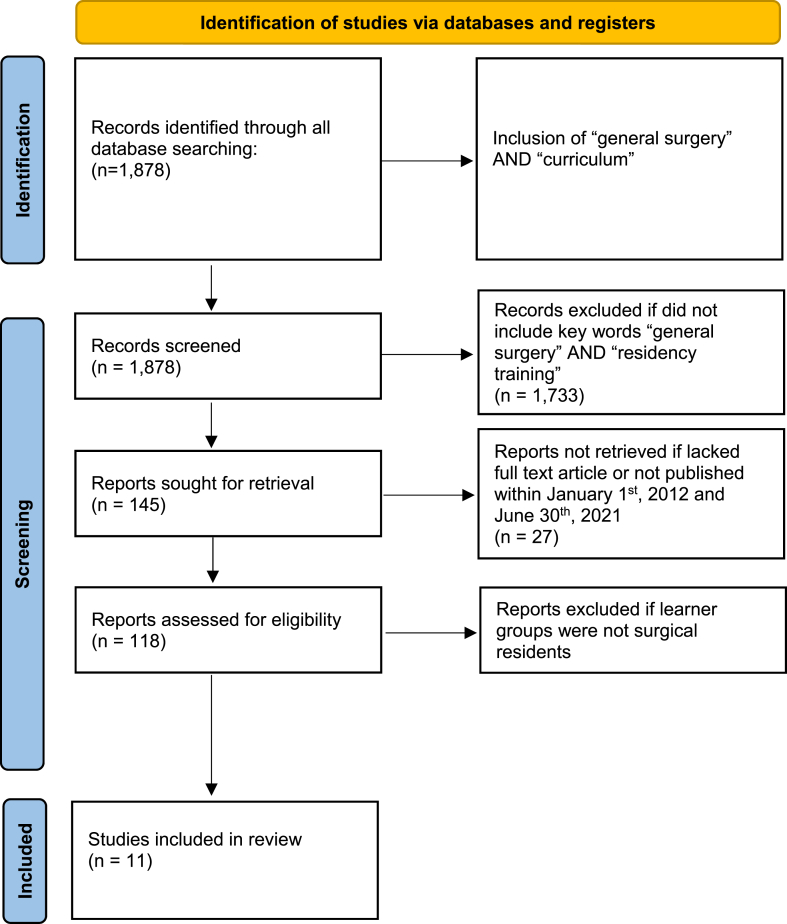
PRISMA protocol literature search results. PRISMA, Preferred Reporting Items for Systematic Reviews and Meta-Analyses [6].

We conducted a PubMed/MEDLINE search to assess and collect available literature on robotic surgery curriculum programs for surgical trainees (Fig. 1). We used the search terms “robotic surgery” [AND] “curriculum”, which identified 1878 results. This was further narrowed to 145 articles with the term “general surgery” [AND] “residency training”. Full texts were available for 138 articles. We also constricted our search to within the last 10 years of publications and 118 full text articles were left to review. Our inclusion criteria therefore included: English language articles, published between January 1, 2012 and June 30, 2021, described curriculum or curricula, and recipient targets of surgical trainees. Based on abstract and titles, the final number of articles reviewed with assessment of robotic surgery curriculums within a residency program were 11. Date of publication ranged from 2014 to 2021. The search included reviews, randomized controlled trials, cohort studies, and systematic reviews. Further inclusion terms that were assessed for in choosing the final 11 articles were that they included training programs, resident learning groups, robotic surgical platform, and subjective or objective outcome measures. Exclusion criteria consisted of articles without full length accessibility, non-English language articles, or non-surgical resident learner groups. Primary outcomes analyzed were subjective data in the form of surveys and objective data from simulator metrics. Secondary data analysis evaluation included curriculum aspects. Data extraction included first author last name, year of publication, journal of publication, study type, learner group, surgical procedures performed for evaluation, form of curriculum assessment, and major conclusions made from the data.

## 3 Results

We reviewed 11 studies that ranged from the years 2014–2021 (Table 1). Of our 11 studies, 64% were retrospective reviews. Two studies were randomized trials. The learner group in 55% of the studies was General Surgery residents. The others included OBGYN and Urology resident learner groups. Two studies reviewed surgical specialties as a whole and therefore included various groups. The surgical procedure used to evaluate learner groups was varied and depended on the learner group specialty. General surgery learner groups most commonly performed cholecystectomies or hernia repairs as their index case while Urology groups used prostatectomy and OBGYN groups used hysterectomy. Forty-five percent of the studies used single index cases for evaluation. The rest of the studies were variable, using aspects such as general surgery basic skills, all general surgery cases performed, or important aspects of robotic training like emergency undocking. The form of assessment was inconsistent and included a range from exclusively subjective surveys to objective simulator metrics. The older studies (2014–2015) used conventional intra- and peri-operative metrics such as operative time and complication rates. Then there was a progression to subjective questionnaires made by programs themselves which included resident participation experience and confidence perception assessments. Standardized metrics such as GEARS, NTLX, and RO-SCORE were incorporated in 2019. These standardized metrics included the teacher (RO-SCORE, GEARS) and learner (NTLX) perspective of the experience. Finally in 2021, case logging and objective simulator metrics started to be used and is seen in one of our 11 studies. Curriculum aspects were also varied but robotic simulators were one universal aspect. This was seen in 100% of the papers reviewed. The next most common aspect was online modules in 45% of the studies. Labs, including dry and wet labs, was seen in 36% of the study curriculums reviewed. Dedicated one-on-one or faculty led teaching sessions was seen in 36% of the studies.

**Table 1:** Summary of articles used in the literature review. GS – General Surgery, MC – multiple choice, GEARS – Global Evaluative Assessment of Robotic Skills, RO-SCORE – Robotic Ottawa Surgical Competency Operating Room Evaluation, NTLX – NASA Task Load Index, RALS – Robotic Assisted Laparoscopic Surgery.

First Author	Year	Journal	Study Type	Learner Group	Surgical Procedure	Assessment	Curriculum Aspects	Conclusions
Juza [7]	2014	Journal of Robotic Surgery	Retrospective Review	General Surgery residents	Robotic cholecystectomy	Operative time, length of stay, complications	Online modules Robotic simulator	No change in metrics pre and post introduction of robotic systems and trainee integration into curriculum. It is safe to integrate robotics
Ayloo [8]	2014	International Journal of Medical Robotics and Computer Assisted Surgery	Retrospective Review	General Surgery residents	Robotic cholecystectomy	Operative time, length of stay, complications	Dry-lab with robotic trainer Animal lab	Complication rates during the introduction of robotics to residents are comparable to rates in laparoscopic cholecystectomy
Morgan [9]	2015	World Journal of Urology	Retrospective Review	Urology residents	Prostatectomy	Intraoperative details Complication rates	Dual-console implementation One-on-one teaching	Dual-console robotic training leads to decreased mean operative time and complications
Tom [5]	2017	American Journal of Surgery	Retrospective Review	General Surgery residents	GS cases (Hernia, CRS, Biliary, Gastric)	13-question survey to GS programs	Robotic Simulator Faculty-directed console time Case Observation	We need further implementation of standardized robotic curricula
Mustafa [10]	2018	Journal of Surgical Education	Retrospective Review	General Surgery residents	Hernia	Trend of resident participation in robotic cases	Online training modules One-on-one bedside teaching session by industry representatives Robotic Simulator	Implementation of curricula means more resident involvement
Ballas [11]	2019	Cureus Journal of Medical Science	Randomized Trial	OB/GYN residents	Emergency Undocking	Survey and MC knowledge questionnaire	Simulation Didactic material Review sessions	Curriculum leads to higher baseline knowledge, confidence, and improvement in undocking times
Moit [12]	2019	Journal of the Society of Laparoscopic and Robotic Surgeons	Retrospective Review	General Surgery residents	GS cases	GEARS	Reading material Online training modules Robotic simulator Dry lab	With a structured robotic curriculum, residents can obtain the skills to operate robotically without an MIS fellowship
Satava [13]	2020	Annals of Surgery	Randomized Trial	Surgical Specialties (residents, fellows, attendings)	Basic Skills (knot tying, ring transfer, etc.)	44-question MC test standardized checklist video reviewing GEARS scale	Online modules Cognitive test Dry lab Animal lab	FRS curriculum is effective
Gerull [14]	2020	Surgical Endoscopy	Non-randomized trial	Surgery residents (various surgical specialties)	RALS case	RO SCORE and NTLX	Robotic simulator modules	Self-directed simulation can translate skills to the clinical environment
Grannan [15]	2021	Surgical Laparoscopy Endoscopy& Percutaneous Techniques	Retrospective Review	General Surgery residents	GS cases	Surveys Case Logs	Online modules Dry lab Robotic simulator	Increased number of operative cases and active engagement with the use of simulation
Turner [16]	2021	Journal of Gynecology Oncology	Cohort Study	OB/GYN residents	Robotic Hysterectomy	Simulator metrics (arm collision, camera distance, number of instrument clutches)	Robotic simulator One-on-one teaching	Standardized 30min sessions q4mo in which procedural steps are reviewed with one-on-one coaching led to increasing proficiency through a robotic hysterectomy simulation

## 4 Discussion

The introduction of anything new always presents itself with challenges of implementation, as well as challenges in the adoption of a changing model. Hospital systems are extremely complex and even small changes have a multitude of implications. That said, it is important to note that there have been studies to show that the implementation of a robotics curriculum and resident training of robotic systems is safe [6 7]. An analysis of surgical complications, length of stays, and operative times shows no significant difference between pre- and post-implementation of a program. Therefore, if we ascertain that it is safe and we know that most agree that training in this new surgical arena is vital then it becomes important to address the aspects of standardizing competency assessment within robotic surgery.

Given this transition within our field, there have been various efforts to create and expand the training platforms. Thus far, there has been no uniform method of training and platforms vary from web-based to on-site and simulators [17]. One of the web-based training curricula that exists is the “Fundamentals of Robotic Surgery” funded by a grant from the Department of Defense and Intuitive Surgical. It is a curriculum that was developed by a cohort of 80 experts, and it includes 4 online training modules consisting of didactic instruction, psychomotor skill development and team training. Their goal is to provide an initial accreditation and they do not intend their program to be used as a full resident training program. Another online training program is one offered by Intuitive Surgical themselves named “Technology Training Pathway” which includes modules regarding product training and skills application. While it is evident that our world continues to shift towards online learning, there will always be invaluable need for on-site training. SAGES has developed a Robotics Masters Series. This program aims to provide educational resources for different levels of expertise from resident physicians to experienced surgeons. They use their annual meeting platform and robotic cadaver courses to integrate on-site aspects to their curricula. Another on-site based curriculum is the Robotics Training Network (RTN) curriculum in which phases of training are guided. The first phase constitutes bedside assists with self-guided learning via online modules for eventual transition to the operating room. Assessment is performed with the Robotic-Objective Structured Assessment of Technical Skills (*R*-OSATS). RTN has now been integrated at 50 programs nation-wide. Lastly, the Fundamental Skills of Robotic-Assisted Surgery (FSRS) Training Program is a two-day to three-week on-site program in New York that allows for various levels of certification (basic, intermediate, and advanced). It uses online modules, labs, and simulators to teach and certify healthcare providers and it is available to all surgical staff including surgeons, nurses, and technicians. Finally, several surgical robotic training simulators exist. The first simulator made available was the dV-trainer with now over 60 training exercises and a range from basic to advanced skills training. Programs themselves, such as FSRS, have developed their own simulators. The Robotic Surgery Simulator (RoSS) is a stand-alone robotic surgery simulator developed by FSRS. One unique aspect of this simulator has been the employment of haptics to provide feedback. Another stand-alone trainer and the most recent addition has been the RobotiX Mentor. Finally, the da Vinci Skills Simulator (dVSS) by Intuitive Surgical has the major benefit of using the actual robotic surgery console that is used in the operating room therefore facilitating familiarization with the system. As it is evident here, there is great variety of training modules and as it is evident in our review there has been much variety in what programs have chosen to implement.

Our review therefore makes evident two important concepts-there is great variety in training curriculums and there is great benefit in implementation. As was notable in just the eleven studies that we used for our systematic review, there is variability in the specialties using robotic technology therefore different cases and skills that are deemed of interest. This has led to different incorporations of curriculum aspects such as some programs using more prep time with online modules and dry labs while others have focused on simulator tasks. Despite these great differences, we come across the unifying factor and second evident concept of our review-there is great benefit in curriculum implementation. The conclusions made by the different studies in our review have shown reduced operative times, greater resident involvement, higher confidence perception, and increased proficiency with curriculum execution. Undoubtedly, the variation did not change the value. For example, Mustafa et al. showed in their study that resident involvement in robotic surgery increased across all PGY levels when comparing before-robotic and after-robotic curriculum. This is useful and a positive outcome of simulator training given that it allows for learning of skills within realistic simulation prior to the operating room, increasing learner and faculty confidence in capability. The benefit of implementation has been acknowledged by residents and attending staff alike from multiple subspecialties, as in evident in a survey study performed by Carroll et al. in which 100% of residents surveyed believed a formal curriculum should be implemented within their respective programs [18]. Similarly, an updated survey of program directors across the country showed agreement in 83% of respondents that robotic-assisted surgery training provided a net benefit to general surgery residents [19].

As mentioned, while it is evident that there are a great variety of tools for robotic-surgery training, how many surgical resident training programs are using them? A 2018 question survey sent to the 277 Accreditation Council for Graduate Medical Education (ACGME) program administrators found a 41% response rate and of the responding programs, 68% had a robotics curriculum [5]. Interestingly, 92% of responding programs noted that their residents are involved in robotic-assisted surgeries and 96% believe that robotic surgery is important for residents. This same study evaluated the most common features of curricula used in the programs and found that 99% used a robotic simulator and 52% used online courses. Only 45% used industry-sponsored training courses and 17% used live animal labs. From all this information we can gather that although there are many tools, we have not found a way to have all resident training programs involved and we do not have a standardized way to train and assess robotic-assisted surgery competency. Sridhar et al. arrives at this same conclusion and goes one step further to propose a standardized approach [20]. It starts with online modules followed by procedure video learning and hands-on docking training. Then, after assessment of competency, the simulator can be used for the development of basic and advanced tasks. Progression can then be made to a dry lab simulation followed by a wet lab. Along the way the proposition is to have assessment and testing benchmarks prior to proceeding. Finally, the trainee can move towards modular training in the operating room. This structure allows for a framework.

Since the incorporation of robotic surgery into the general surgery arena and since the implementation into surgery training there has been a slow but steady rise of inclusion. From our review of the literature we can see that programs that chose to structure their curriculum and assess their changes have found positive results. At first, through the use of conventional operative metrics such as operative times and complication rates, programs were able to quantify that there was safe integration of robotic platforms. Once that was established, there was assessment of how the integration of such programs benefited the residents. The studies that we reviewed showed that subjective confidence in their capability and objective baseline knowledge also improved with structured training. Standardized assessment tools such as GEARS helped to demonstrate skill acquisition over time therefore assessing development throughout training. If nothing else, this is an important part of tracking progress [12]. Similarly, there was a study that used RO-SCORE (a modified O-SCORE) and the NTLX. The value in this study is that they used a structured approach to learner and teacher evaluation. Uniquely, the NTLX survey quantifies mental and physical demand, as well as effort and frustration. The importance, again, of these structured approaches to assessment is the utility of assessing development and change over time. Finally, methods of recognition for robotic skills expertise are variable within programs with most using a certificate from industry or the residency program, but others also using case logs [5]. This is an important aspect of curriculums as this often becomes the sole outcome measure that residents can show to account for their proficiency and expertise in validation of the safe practice of robotic surgery as they move forward after surgical training. A comprehensive discussion on the budget required and the value added to implement a robotics curriculum is outside the scope of this review. However, given the expense of purchasing a Da Vinci system and the mission of an academic surgical residency program to ensure the competency of its graduates, the implementation of a robotic training curriculum seems to be a necessary and relevant expense.

The conclusion that can be made from this review article is that programs vary widely in the aspects that they choose to incorporate into their programs-whether it be due to different time constraints. Different resident learning styles or different subspecialties altogether. These different demands make for different curriculums and the use of contrasting assessment tools. In this review we do not aim to propose another standard of what a training curriculum should be because that would only add to the variations that exist now. What we need to focus on is the important aspects of the curriculum that allow for scalability of training. Training within resident education is a model and robotic technology is just one of its many uses. Forty years ago, there was the introduction of laparoscopy, now there is robotic technology, and there will surely be future introductions to our field. With that said, the importance is in establishment of what makes resident training effective and supports the adaptable and successful surgeon. This may come from an adaptable curriculum but a structured test-out assessment.

## Author contribution statement

All authors listed have significantly contributed to the development and the writing of this article.

## Data availability statement

Data included in article/supp. Material/referenced in article.

## Additional information

No additional information is available for this paper.

## Declaration of competing interest

The authors declare the following financial interests/personal relationships which may be considered as potential competing interests:Haydee del Calvo, MD – No disclosures, Min P. Kim, MD – Medtronic, Intuitive Surgical, Olympus (Speaking/Teaching) and AstraZeneca (Consultant), Ray Chihara, MD PhD – No disclosures, Edward Y. Chan – Intuitive Surgical and Olympus (Consultant).
